# Liver injury following blunt abdominal trauma: a new mechanism-driven classification

**DOI:** 10.1007/s00595-013-0515-7

**Published:** 2013-03-05

**Authors:** J. E. Slotta, C. Justinger, O. Kollmar, C. Kollmar, T. Schäfer, M. K. Schilling

**Affiliations:** 1 Department of General Surgery, Visceral, Vascular and Paediatric Surgery, University of Saarland, Homburg/Saar, 66421 Saarland, Germany; 2 Department of Trauma, Hand and Reconstructive Surgery, University of Saarland, Homburg/Saar, 66421 Saarland, Germany

**Keywords:** Liver injury, Blunt abdominal trauma, Classification, Trauma mechanism

## Abstract

**Purposes:**

The current classifications for blunt liver trauma focus only on the extent of liver injury. However, these scores are independent from the localization of liver injury and mechanism of trauma.

**Methods:**

The type of liver injury after blunt abdominal trauma was newly classified as type A when it was along the falciform ligament with involvement of segments IVa/b, III, or II, and type B when there was involvement of segments V–VIII. With the use of a prospectively established database, the clinical, perioperative, and outcome data were analyzed regarding the trauma mechanism, as well as the radiological and intraoperative findings.

**Results:**

In 64 patients, the type of liver injury following blunt abdominal trauma was clearly linked with the mechanism of trauma: type A injuries (*n* = 28) were associated with a frontal trauma, whereas type B injuries (*n* = 36) were found after complex trauma mechanisms. The demographic data, mortality, ICU stay, and hospital stay showed no significant differences between the two groups. Interestingly, all patients with type A ruptures required immediate surgical intervention, whereas six patients (16.7 %) with type B ruptures could be managed conservatively.

**Conclusions:**

This new classification for blunt traumatic hepatic injury is based on the localization of parenchymal disruption and correlates with the mechanism of trauma. The type of liver injury correlated with the necessity for surgical therapy.

## Introduction

In Western countries, blunt liver injuries are caused by traffic accidents in approximately 70 % of cases [1]. In the case of polytraumatized patients with open or blunt abdominal trauma, the liver is the most frequently injured abdominal organ [2–4]. Thirty-one percent of polytrauma patients have abdominal injuries, and lesions to the liver are found in 16 % of patients [5]. The main cause of liver injury-related death is uncontrolled bleeding, and it is associated with a mortality rate of 54 % [6].

However, the management of traumatic liver injuries has changed during recent years, and the outcome of patients has markedly improved [3, 7, 8]. Surgical treatment was the standard procedure for all kinds of trauma-related liver injuries, based on the idea that surgery was necessary to control the bleeding and prevent biliary complications. However, an improved understanding of the natural course of liver injuries and the development of new interventional radiological techniques have changed the paradigm toward a more non-surgical patient management [8–10]. In the literature, more than 80 % of patients with blunt hepatic trauma are treated in a non-surgical fashion [7, 11, 12].

In addition, the comprehensive introduction and use of CT scanning enabled a reliable diagnosis of liver injuries within a short time after admission to the emergency room, and has become the gold standard for assessing trauma patients [4]. CT scan-based classifications of liver injuries [13] allowed for the selection of patients who could be managed conservatively. A prerequisite for a non-surgical approach is a haemodynamically stable patient with no further need for a laparotomy [11]. This conservative approach, however, should only be used in centers with an appropriate infrastructure providing capabilities for intensive care monitoring and instantaneous surgery [12]. However, these centers do not necessarily have to be high volume centers [11].

A variety of classifications for traumatic liver injuries have been described in the literature. The most accepted scoring system is the Moore score [14], which is based on the Organ Injury Scale (OIS) of the American Association for Surgery of Trauma (AAST) which was published in 1989 [15]. The Moore score is considered a gold standard to describe liver injuries. Another well-established scoring system is the Mirvis score [13], which is based on CT-graphic findings and gives the first hints about the necessity of surgery for patients with traumatic liver injuries.

However, the current scoring systems do not incorporate the localization of liver injury or the mechanism of trauma. Therefore, we developed a new classification for liver injuries and analyzed our patient cohort regarding the mechanism of trauma and corresponding pattern of liver injury.

## Patients and methods

### Data acquisition

Between January 2000 and February 2011, all patients admitted to our emergency room following blunt abdominal trauma were routinely screened for liver rupture. All patients diagnosed with liver injury either by CT scan or intraoperative findings were prospectively entered in an i.s.h.-med database (GSD, Berlin, Germany) running on a SAP platform (SAP, St Leon-Rot, Germany). The demographic, peri-, and postoperative data, as well as patient outcome, were analyzed retrospectively.

### Patient management

Patients were admitted to the emergency room of our major trauma center. The trauma surgeon on call performed the first physical examination, including abdominal ultrasound. In haemodynamically stable patients, CT scanning was performed to evaluate the extent of injury. Further procedures were dependent on the CT findings. Critical and unstable patients as well as initially stable patients who became unstable during the diagnostic procedures with sonographic evidence of free intraabdominal fluid were directly transferred to the operating room without further diagnostic procedures for an explorative laparotomy by the visceral surgeon on call. Haemodynamic instability was the sole criterion for immediate surgical treatment, and this criterion was introduced by Kozar in 2009 [16]. Angioembolization, which is also a treatment option for blunt liver trauma with CT-graphic evidence for liver rupture and bleeding, was not performed in this cohort.

The localization and extent of trauma-related injuries were determined by the visceral surgeon during surgery by exploration of the entire abdominal cavity. Surgical procedures were performed based on the intraoperative findings. In cases with liver parenchymal transection, the surgical procedures included suturing of the liver, anatomical or atypical liver resections, as well as liver resections including right or left hemihepatectomy with or without Pringle’s maneuvre. Anatomical or atypical resections were usually performed with different dissection devices, and major resections were performed using linear cutting devices. The use of techniques to achieve haemostasis, such as packing, argon beam, and/or tissue sealants (TachoSil^®^; Nycomed, Konstanz, Germany), was based on the current surgical standards of the department.

After surgery or conservative treatment, which was only performed if patients were haemodynamically stable upon admission to our emergency unit, the patients were transferred to the intensive care unit for resuscitation and therapy. Monitoring of the patients was performed by physical examination, ultrasound, and blood analyses. If necessary, further injuries were treated by the respective specialists.

The localization of liver injury was assessed either by CT scanning, as described by Mirvis, or based on the intraoperative findings. Liver injury was defined as any disintegrity of the liver surface or parenchymal transection within the liver. Attribution to the respective type of liver injury was performed based on the localization of liver injury, whereas the classification we present herein has not been described previously. Both the mechanism of trauma and type of liver injury according to our proposed classification had no influence on the decision of whether to perform surgical or non-surgical management.

### Statistical analyses

The data are expressed as absolute numbers, percentages, or the mean ± SEM unless indicated otherwise. The length of follow-up was calculated from the date of admission to our institution until the time of death or the day of discharge. Differences between the two groups were calculated using Fischer’s exact test, the Mann–Whitney *U* test, or Student’s *t* test, as appropriate. The statistical analyses were performed using the SPSS 18.0^®^ (IBM Deutschland GmbH, Ehningen, Germany) or SigmaStat (Jandel Scientific, Jandel, San Rafael, CA, USA) software package. *p* values <0.05 were considered to be significant.

## Results

### Patient demographics

Between January 2000 and February 2011, sixty-four patients (22 female, 42 male) were admitted to our emergency room with blunt liver trauma. The mean age of the patients was 39.1 ± 2.7 years. Fifty-eight patients required immediate surgery due to haemodynamic instability. The average stay in the intensive care unit was approximately 10 days, with a median hospital stay of 17 days. The mortality rate of all patients was approximately 30 %, and death occurred in 10 out of these 16 patients during the first 2 days after admission, independent of the type of liver injury (Table 1).

**Table 1:** The demographics, length of hospital stay and morbidity and mortality rates of 64 patients with blunt liver injuries

Variable	
*n*	64
Gender (female/male)	22/42
Age (years)	39.1 ± 2.7
Moore score	2.6 ± 0.2
Mirvis score	2.5 ± 0.2
Conservative treatment	6
ICU stay (days)	10.4 ± 1.4
Hospital stay (days)	17.3 ± 1.8
Mortality (%)	29.7

Data are given as *n*, mean ± SEM, or %

### Type of liver injury

**Fig. 1:**
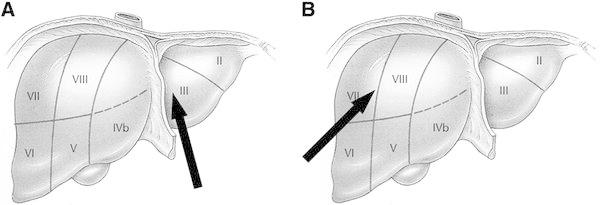
An illustration of vectors of energy impact on the liver. Frontal energy transfer (**a**) leads to type A injuries in the left liver lobe, whereas more complex mechanisms of trauma (**b**) cause type B injuries in the right liver lobe. The *arrows* indicate the direction of the impacting energy

**Fig. 2:**
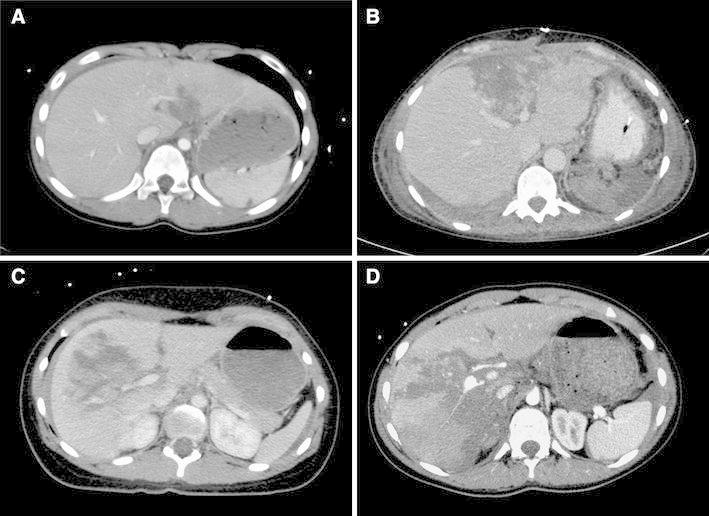
Representative CT scans in axial projections of two patients with type A (**a**, **b**) and two patients with type B (**c**, **d**) injuries following blunt liver trauma. The patient with the type B liver rupture was managed conservatively despite massive destruction of the hepatic parenchyma corresponding to a grade IV injury according to Moore score. The underlying traumas in these patients were **a** fall from a 3 m height onto the abdomen, **b** a rear-end collision, **c** a side-impact car crash, **d** a fall from a 10 m high climbing scaffold

According to the localization of liver injury and the mechanism of the underlying blunt liver trauma, liver rupture could be classified into two types:

A. Type A patients suffered from a rupture of the left liver lobe mostly along the falciform ligament, including segment II, III or IV of the liver (Figs. 1a, 2a, b). This injury pattern was observed when the trauma had a direct frontal impact of the trauma energy (Table 2).

**Table 2:** Trauma mechanisms

Type A	*n* = 28	Type B	*n* = 36
Frontal car accident	15	Complex traffic accident	22
Mechanical cardiopulmonary resuscitation	2	Fall from roof	4
Fall on stairs	3	Suicidal jump	2
Hit by a falling branch	1	Hit by crane	1
Others	7	Stuck between army vehicles	1
		Horse kick	1
		Crash with football player	1
		Others	4
High energy trauma	19/28	High energy trauma	32/36
B. Type B injury represented mechanisms of trauma with a more complex pattern of energy, with impacts coming from several directions (Table 2), affecting segments V–VIII of the liver (Figs. 1b, 2c, d).

Examples of CT scans from patients with each type of trauma are shown in Fig. 2 (Type A in Fig. 2a, b; and type B in Fig. 2c, d). Liver rupture was most commonly accompanied by additional injuries, including multiple affected organs and the musculoskeletal system. The affected organs are listed in Table 2. Comparing the liver injuries in both groups, patients with type B liver ruptures had more additional injuries, such as bone fractures and thoracic injuries, than patients with type A liver ruptures (Table 3). However, additional bone fractures or thoracic injuries were not the risk factors for the type of liver injury, as assessed by a multiple logistic regression analysis [thoracic: OR 1.180 (0.306–4.543 95 % CI); bone: OR 1.937 (0.509–7.371 95 %CI)].

**Table 3:** Additional injuries of patients with type A (*n* = 28) and type B (*n* = 36) liver ruptures listed as the affected organ

Affected organ	Type A	Type B	*p* value
Bone fracture	15 (54 %)	32 (89 %)	<0.001
Thorax (incl. hematothorax, pneumothorax, serial costal fracture, cardiac contusion)	12 (43 %)	26 (72 %)	0.023
Head/face	9 (32 %)	16 (44 %)	0.310
Lung	11 (39 %)	22 (61 %)	0.130
Kidney	2 (7 %)	6 (17 %)	0.282
Spleen	13 (46 %)	11 (31 %)	0.298
Stomach	4 (14 %)	1 (3 %)	0.162
Bowel	5 (18 %)	7 (19 %)	1.000
Pancreas	7 (25 %)	8 (22 %)	1.000
Aorta	2 (7 %)	2 (6 %)	1.000
Soft tissue	12 (43 %)	23 (64 %)	0.303
Brain	14 (50 %)	18 (50 %)	1.000

Data are given as *n* as well as percentages

### Patient demographics according to the type of liver injury

An analysis of the demographic data showed no significant differences between patients with type A and type B liver trauma. Patients with type B injuries had higher Moore and Mirvis scores (Table 4) indicating more severe trauma compared to patients with type A liver injuries, but the difference did not reach statistical significance. Interestingly, all patients with type A injuries required immediate surgical treatment for bleeding control due to haemodyamic instability, whereas six out of the 36 patients with a type B injuries were treated with a conservative, “watchful waiting” approach, without increased mortality (*p* > 0.05). Further subgroup analyses revealed that neither gender nor age were risk factors for death following liver rupture due to blunt abdominal trauma, independent of the type of liver injury.

**Table 4:** The demographics, length of hospital stay, and morbidity and mortality rates of 64 patients treated with blunt liver injuries, stratified for patients with type A (*n* = 28) or type B (*n* = 36) liver injuries

Variable	Type A	Type B	*p* value
Gender (female/male)	9/19	13/23	0.796
Age (years)	39.0 ± 3.8	39.2 ± 3.8	0.860
Moore score	2.15 ± 0.18	2.97 ± 0.29	0.071
Mirvis score	2.19 ± 0.21	2.83 ± 0.24	0.084
Conservative treatment	0	6	0.031
ICU stay (days)	10.9 ± 2.4	10.1 ± 1.6	0.978
Hospital stay (days)	18.2 ± 2.9	16.5 ± 2.4	0.927
Mortality (%)	25.0	33.3	0.573
Morbidity (%)	25 %	26.7 %	0.926

Data are given as *n*, the mean ± SEM, or %

## Discussion

Based on the results of this retrospective analysis, a new classification for blunt liver injuries was presented. The new classification is based on the localization of liver disruption by CT scanning or intraoperative findings, and correlates with the mechanism of trauma. Our analyses show that type A injuries resulted from a frontal impact of energy, e.g., in cases of frontal car accidents. This kind of energy transfer causes severe injury of the left liver lobe, i.e., segments II, III, IVa, and IVb. Therefore, type A injuries develop along the falciform ligament. Interestingly, all patients in our analysis who had a type A liver injury required immediate surgery due to haemodynamic instability. Despite immediate laparotomy for bleeding control, type A injuries were associated with a 25 % mortality rate, independent of the severity of liver injury, as assessed by the Moore score or Mirvis score. According to the literature, gender or age were also not the risk factors for type A liver injury-associated mortality [4].

In contrast, type B injuries resulted from more complex mechanisms of trauma, with an impact of energy from other directions than straight frontal, such as due to horse kicks, being crushed between vehicles or a high speed collision. Type B injuries are—according to our new classification—localized in the right liver lobe, i.e., segments V–VIII. Interestingly, the mortality of patients with type B injuries was not significantly different from that of patients with type A injuries, although type B injuries were associated with more complex trauma and more concomitant injuries. Again, gender, age, and the severity of liver injury were not determinants for type B injury-associated death. In contrast to type A injuries, significantly, more patients with type B liver rupture (six out of 36 patients) were haemodynamically stable and survived without surgery. The severity of liver injury in these six patients was not significantly different from that of patients undergoing surgery for type B liver rupture. Due to the higher energy transfer, additional injuries were more frequently observed after type B than after type A liver rupture.

Certainly, the localization of liver rupture, i.e., type A or type B rupture can never be the sole parameter used to decide on whether surgical or conservative management should be used for the liver rupture. Instead, the decision should be made based on the haemodynamic stability and lack of other injuries requiring abdominal surgery. To what extent the localization of liver disruption, and thus, the type of liver injury according to our new classification, will contribute to the decision to perform surgical or conservative management cannot be answered based on our data presented herein.

Irrespective of the type of liver injury, there has been a trend toward a more conservative and “watchful waiting” management of patients during the past two decades [5, 7, 11, 17]. Several studies have demonstrated that up to 80 % of the patients with blunt liver trauma can be managed conservatively [11], but these reports do not nominate other factors than the haemodynamic stability for making the decision on whether to use a surgical or non-surgical approach. In our study, only 9.4 % (6/64) of the patients could be managed conservatively within the last 11 years. During this time, the treatment strategies have changed, and angioembolization has been introduced for the treatment of blunt liver injuries and has been proven to be an effective treatment option even for patients with severe liver injuries [18, 19]. Since this technique has not been performed during the observation period at our center, our data include those of patients who could likely have been treated conservatively with angioembolization but underwent surgery due to the unavailability of this technique, which may explain the high percentage of patients requiring surgical therapy.

The current classifications for liver rupture are the Organ Injury Scale proposed by the American Association for Surgery of trauma (AAST-OIS) [20] or the Moore score [14, 15] and the Mirvis score, which describe the extent of liver damage either morphologically (AAST-OIS) or based on CT findings [13]. These scaling systems are widely accepted due to their long-term use, and in the case of the Mirvis score, due to the comprehensive use of CT scanning for trauma diagnostics. In addition, these scoring systems are validated and offer clear prognoses for different grades of injury severity [20]. However, these scoring systems do not incorporate the localization of liver injury, i.e., left or right liver lobe, or the mechanism of trauma and vector of power impact. One might argue that the site of the liver or direction of impact is irrelevant when making the decision whether to perform a laparotomy in a patient with blunt liver rupture or to choose a conservative approach. While this is somewhat reasonable, since haemodynamic stability, additional injuries, and other factors confound the decision, our data clearly demonstrate that more complex trauma mechanisms mainly affect the right liver lobe. Injuries in this liver lobe can be better tolerated, in the sense that they do not necessarily cause haemodynamic instability and are more often self-limiting. This knowledge might allow for the choice of a conservative treatment approach without an increased risk of mortality. Nevertheless, the localization of liver disruption alone can never be the sole parameter for deciding on surgical or conservative treatment.

As a possible explanation for the more severe injury in Type A cases, we suppose that a frontal energy impact first leads to an acceleration of the liver toward the spine. When the liver is decelerated by the falciform ligament, which is very stable and rigid, the liver is disrupted along this ligament, and large intrahepatic vessels are destroyed leading to severe bleeding toward the abdominal cavity. In contrast, the large volume of the right liver lobe and the direct covering of the right liver by the diaphragm (area nuda) allow the right liver to compress and self-limit even a severe disruption of large blood vessels.

In conclusion, we herein propose an additional classification that can be used in combination with the current and established scoring systems and treatment algorithms for blunt liver injuries, which is based on the localization of the liver injury and which represents the mechanism of blunt liver trauma. According to our new classification, type A injuries, which occur along the falciform ligament, are associated with a relevant haemodynamic instability requiring immediate surgical therapy. Type B injuries involving the right liver lobe are more likely to be self-limiting, and a watchful waiting strategy is justified if there are no other factors requiring abdominal surgery. Importantly, this classification is neither a decision aid nor a treatment algorithm with regard to surgical or non-surgical management, nor is it capable of predicting the outcome or mortality. Since our data were obtained from a retrospective analysis, a prospective validation and evaluation of the practicability of this classification for the emergency room is necessary.
